# Taking Advantage of Selective Change Driven Processing for 3D Scanning

**DOI:** 10.3390/s131013143

**Published:** 2013-09-27

**Authors:** Francisco Vegara, Pedro Zuccarello, Jose A. Boluda, Fernando Pardo

**Affiliations:** 1 Departament d'Informàtica, Escola Tècnica Superior d'Enginyeria, Universitat de València, Avd. de la Universidad, s/n, Burjassot, València 46100, Spain; E-Mails: Francisco.Vegara@uv.es (F.V.); Jose.A.Boluda@uv.es (J.A.B.); 2 Instituto de Microelectrónica de Barcelona, IMB-CNM (CSIC), Campus Universitat Autónoma de Barcelona, Bellaterra, Barcelona 08193, Spain; E-Mail: Pedro.Zuccarello@imb-cnm.csic.es

**Keywords:** event-based vision, high-speed visual acquisition, 3D scanning

## Abstract

This article deals with the application of the principles of SCD (Selective Change Driven) vision to 3D laser scanning. Two experimental sets have been implemented: one with a classical CMOS (Complementary Metal-Oxide Semiconductor) sensor, and the other one with a recently developed CMOS SCD sensor for comparative purposes, both using the technique known as Active Triangulation. An SCD sensor only delivers the pixels that have changed most, ordered by the magnitude of their change since their last readout. The 3D scanning method is based on the systematic search through the entire image to detect pixels that exceed a certain threshold, showing the SCD approach to be ideal for this application. Several experiments for both capturing strategies have been performed to try to find the limitations in high speed acquisition/processing. The classical approach is limited by the sequential array acquisition, as predicted by the Nyquist–Shannon sampling theorem, and this has been experimentally demonstrated in the case of a rotating helix. These limitations are overcome by the SCD 3D scanning prototype achieving a significantly higher performance. The aim of this article is to compare both capturing strategies in terms of performance in the time and frequency domains, so they share all the static characteristics including resolution, 3D scanning method, *etc.*, thus yielding the same 3D reconstruction in static scenes.

## 1. Introduction

A 3D scanner is a device typically used for measuring distances without physical contact in a systematic way. These devices have been developed to solve specific problems in a vast range of fields, for instance in industrial inspection, robotics or architecture. To overcome the drawback of the high cost associated to these devices, many alternative designs have emerged.

There are several technological solutions for measuring distances with these devices: photogrammetry, interferometry or time-of-flight. The most commonly used technique is known as Active Triangulation. This method is relatively easy to implement, giving good enough results for measuring distances in the range of several meters to μm. These kinds of scanners usually consist of an element that generates a light pattern (typically a laser line generator) and an element that records the pattern projected onto the surface to be measured, usually by means of a CCD (Charge-Coupled Device) or a CMOS camera.

Like any other measurement technique, Active Triangulation has several error sources that limit its resolution. A good approach to this ancient technique, and an analysis of its restrictions, can be seen in [1]. Additionally, over time many contributions trying to overcome these and other drawbacks have appeared. For instance, in [2] the authors propose three techniques to reduce errors incurred in measurements of triangulation sensors. Particularly, for the case when the object is not perpendicular to the incident light, and when the characteristics of the object surface vary. The merits and usefulness of the three techniques are discussed, enabling choices to be made with respect to cost, size, speed or error reduction. The third proposed method implies the use of several sensors, viewing from different directions, and a statistical method to obtain a robust average of all sensor signals. Other authors propose solutions to increase the accuracy of measurements through sub-pixel resolution [3]. Therefore this method should be applied for noise free images because it is heavily noise dependent. Similarly, there are super-resolution techniques for improving the resolution of surfaces captured with a laser scanner by combining many similar scans [4]. These techniques are much more time consuming, but can be useful in high-value surface scans, when time is not a critical factor. On the other hand, sometimes the basic configuration is changed in order to minimize the occlusion problem due to laser/receiver separation. Typically this problem can be solved if two cameras are used, or alternatively as shown in [5], a system with two different colored lasers and a single CCD camera. Another solution that tries to reduce the same problem can be seen in [6], where the configuration adopted in this case is a central laser and two lateral CCD cameras that record the surface of the objects from different perspectives. If we compare this system with a scanner which uses two lasers and one camera, the two-laser scanner has benefits such as a lower price, less complexity, and lower computing to prepare a 3D image.

Despite the large number of contributions in this field, there are not so many developments that focus on improving the detection system technology itself. One of these contributions is BIRIS [7] sensor for distance measurement, developed in the early 90 s, where the whole system is called and treated as a single sensor. In fact, this system can be considered one of the first classic scanner prototypes with applications to mobile robotics. The BIRIS system consists of a sensor head and processing system. The head assembly holds a CCD camera and laser line projector. A mask within the camera lens area creates a double image. The separation of the imaged lines and the center position between the lines is a function of the range. The processing system captures the images, detects peaks and calculates the range points. Rather than develop a complete set of formulas for a theoretical model, the authors have used the principles of operation to develop tests which assess the sensor's performance for given mobile robot tasks. That work illustrates the need for thorough testing when dealing with a complex system.

Further developments have achieved small enough commercial sensors which are useful in robotic applications, such as for example the Hokuyo URG-04LX sensor, or the SICK LMS 200 which is very similar, although slightly bigger. The Hokuyo scanner works differently from the SICK sensor that is based on the ToF (Time-of-Flight) measurement principle. It uses amplitude modulated laser light and deduces the target distance from the phase shift measurement between the emitted light wave and its reflection. In [8] there is an in-depth comparison of both sensors. Results for the Hokuyo sensor show that sensor accuracy is strongly dependent on the target surface properties, consequently it is difficult to establish a calibration model.

There are also other commercial products using the ToF technique, such as the MESA SR4000 scanner sensor with a resolution of 176 × 144 pixels and 50 fps. It is possible to achieve with this sensor pose estimation accuracy as good as that achieved with a stereovision-based approach [9]. Similarly, PMD's CamCube 3.0 uses the same measurement technique and 40 fps for a resolution of 200 × 200 pixels, or 80 fps for 160 × 120 pixels. In [10] a comparative study of two laser scanners is shown, the Sick LMS200 and the Hokuyo URG-04LX, for measurement drift over time, the effect of material and color on measurement accuracy, and the ability to map different surface patterns. An in-depth analysis of the state-of-the art in the field of lock-in ToF cameras can be seen in [11]. The performance of ToF range cameras has improved significantly over the last few years; error sources have been reduced and higher resolution and frame rates are being obtained. Despite these improvements, ToF cameras cannot yet achieve the depth accuracy offered by the classical triangulation system. Even there have been developed scanning particle systems to measure the three-dimensional distribution of three-component velocity in a turbulent round jet [12]. Finally, as a last example, in [13,14] a complete analysis, both in terms of accuracy and resolution, of Microsoft's Kinect is shown, a system that can be used in not very demanding indoor applications.

The proposed scanner in this paper tries to take advantage of the SCD sensor, a new event-driven sensor, which delivers only pixels that have changed, ordered by the absolute magnitude of their change. In this way a lot of time can be saved since redundant information is not sent and therefore is not processed. This behavior is especially suitable for the laser scanning problem. The process of selecting the pixels which are illuminated by the laser is automatically solved by the sensor logic at the focal plane. Section 2 describes the SCD sensor and camera.

The main objective of this paper deals with the presentation of the benefits, in terms of performance increase, achieved with an SCD sensor *versus* a classic sensor for measuring distances in a 3D scanner prototype, with the Active Triangulation technique.

An experimental set-up has been implemented, with a rotational movement achieved with a helix attached to a small motor. This movement, or shape change, since this is what the scanner detects, has been used to discover the acquisition speed limits. The experimental setup is described in Section 3, where the principles of the Active Triangulation measuring system are also presented. Section 4 shows experimental and compared results for both systems: the SCD and the classical. Finally, Section 5 draws some conclusions about the obtained results.

## 2. Selective Change-Driven System

An SCD Selective Change-Driven (SCD) system is based on an SCD sensor which has a non-conventional behavior [15]. In each cell of the sensor there is a capacitor that is charged to a voltage during an integration time, like any other image sensor. The first difference in the SCD sensor is that each pixel has its own analogue memory with the last read-out value. The difference between the current and the stored value is compared in parallel for all pixels in the sensor using a Winner-Take-All circuit [16]. The pixel with the greatest change is selected first, and its illumination level and coordinates are read out for processing, the local stored value is updated, so this pixel will lose all next voting until there is, again, a large difference in its illumination level. In the past, and previous to the development of a real SCD sensor, the advantages of this philosophy were demonstrated [17–19]. The first SCD sensor has a resolution of 32 × 32 pixels and, despite its relatively low resolution, it has proved the usefulness of the SCD approach for resource limited systems [20]. All sensor control signals are generated with just a 32-bit 80-MHz PIC microcontroller in a small USB-powered camera. New SCD sensors, with larger resolution and speed, are now being developed.

The main advantage of an SCD camera is that only the information that is new, thus relevant, is sent. Within the SCD philosophy it is not necessary to re-send the pixels that have not changed. They were already sent, and therefore the system should keep track of them. As can be guessed, the design of image processing algorithms within the SCD formalism requires a change in the way of thinking about how the programming instructions are applied to data. Classical processing systems deal with image flow while SCD systems must deal with pixel flow. Most artificial algorithms are based on images and sequences of images, but they can be transformed to work with pixel or event sequences; in fact, most biological systems work in a multiple non-synchronous pixel flow. Nevertheless, it is still possible to have a kind of “image” with information taken from present and past visual information. From the processing point of view, the SCD strategy is related with data-flow architectures: each new pixel fires all the instructions related to this new data. If there are no data changes no instructions are fired, and in this way no time or energy are wasted. In fact, there is no processing of images but of pixels. The system processes pixels instead of images.

Typically an SCD image processing system can be designed as a pipeline of processing stages. Initially the SCD camera delivers those pixels that have changed (sending their grey level and coordinates). The first stage consequently updates the changes of this new pixel to its output. The second and the subsequent stages will also detect that there are new data and they will do the same. When new input data arrives at any stage, all the related instructions are fired, updating the output for each stage. These ideas are explained in greater depth in [21]

Particularly, with this triangulation algorithm it has not been necessary to keep track of the intermediate data due to the experiment characteristics. This experiment tries to show the performance advantages within the SCD-based approach *versus* a conventional scanner. The rotating helix will guarantee changes in the pixels that are contributing to the depth calculation. The next section will explain the experimental set-up and the principles of the Active Triangulation method.

**Figure 1.**
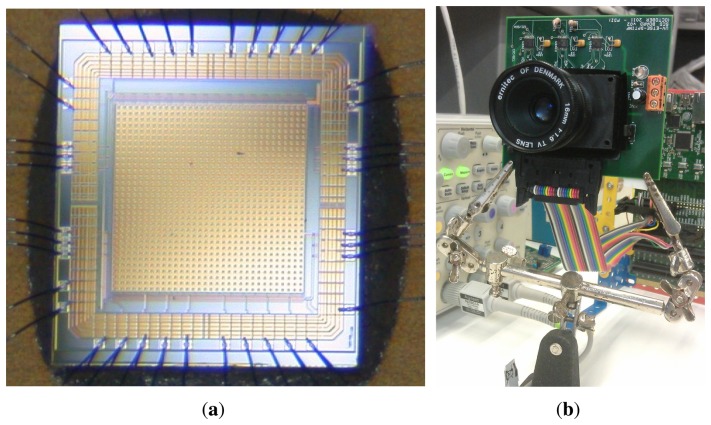
Selective Change Driven (SCD) sensor and camera board. (**a**) SCD sensor; (**b**) SCD camera board.

Figure 1a shows the sensor layout, most of it being covered by a metal layer to protect the pixel circuitry from the light. Only a small window allows the light to reach the photodiode sensing part. Figure 1b shows the SCD camera board developed for the sensor.

## 3. System Description

A scanning system with a removable camera has been implemented in order to compare both possibilities, the SCD approach *versus* the classical. The classical camera is a Firefly MV with a 640 × 480 resolution CMOS monochrome sensor (with adjustable resolution, gain and shutter time). The SCD sensor has programmable acquisition time and number of delivered pixels. For the comparison experiments the resolution window of the first camera was reduced to 32 × 32 pixels, that is, exactly the same resolution as the SCD sensor.

**Figure 2.**
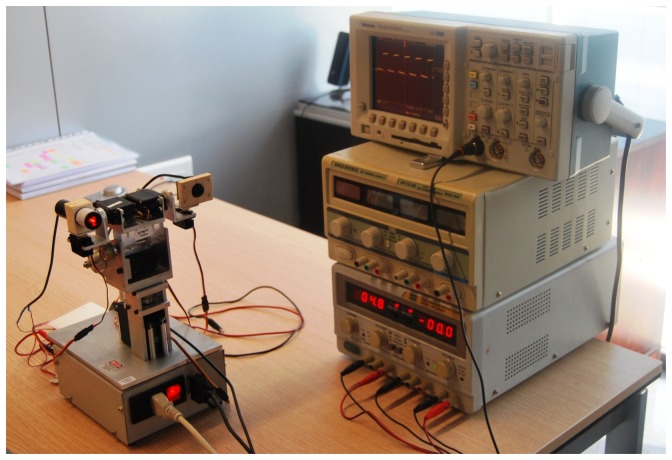
Part of the experimental setup, including the Pan-tilt head with the laser and the Firefly camera.

The system also has a 10 mW, 635 nm red laser that, through a specific lens, generates a linear beam of high-intensity structured light. Both the laser and the camera are mounted on a pan-tilt head with six degrees of freedom, driven by servomotors, where the first two degrees of freedom are the elevation and deviation of the head respectively. The other four degrees of freedom are the elevation and the deviation of both the laser and the camera independently. Figure 2 shows part of the experimental setup, including the pan-tilt head.

The system configuration is fully flexible because both the deflection angles of the laser and the camera can be changed by software. In this way, the detection range can be changed (and hence the resolution, which is reversed) or even the measure direction.

Besides the software resolution selection for the conventional camera, and with the idea of comparing both cameras under the same conditions, the lowest possible shutter time (50 μs) for this camera was chosen. In the same way the greatest possible gain (12 dB) was chosen. Under these conditions, this camera can capture up to 411 frames per second (≈2.43 ms/frame).

### 3.1. Active Triangulation Basics

**Figure 3.**
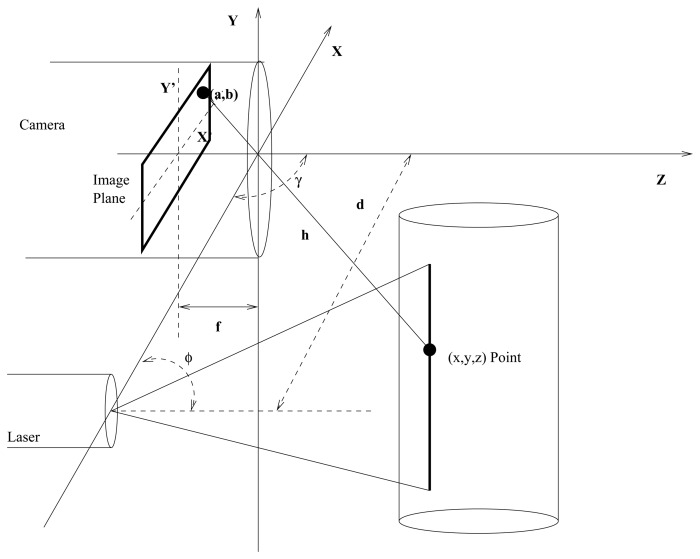
Triangulation scheme.

Active Triangulation is one of the most widely used methods for non-contact distance measurement. There are several calculation methods depending on the objectives. A very common configuration can be seen in [22] or similarly in [13], the basic principle of the method consists of projecting a pattern of light (usually a laser line) on the surface to be measured. Afterwards, the pattern projection is captured in the focal plane of a digital camera. The followed triangulation scheme, where all distances are Euclidean, can be seen in Figure 3.

The distance between the laser source and the camera *d* is known. Additionally, the angle *φ* between the laser and the base line connecting the laser and camera, is also known. Therefore the distance *h* between the camera and the surface illuminated by the laser can be obtained by basic trigonometry (equivalent triangles). This distance is computed measuring displacement in pixels that can be observed in the focal plane with respect to a reference position. An example of this configuration can be seen in Figure 3, where (*a, b*) are the coordinates in the focal plane, and *f* is the camera focal length.

Commonly, the laser points to the front $\left(\phi =\frac{\pi }{2}\right)$ while the focal plane usually changes its angle *γ* with respect to the base line. It can be seen that the smaller the angle between the camera and the laser *γ* + *φ*, the lower the detectable range, but the resolution will be higher. In the proposed setup, it is recommended to use $\phi =\frac{\pi }{2}$ and $\gamma =\frac{\pi }{2}$, for the detection of surfaces up to a relatively large distance (up to 6 m). With this configuration, the spatial coordinates of the target point can be obtained by the Equations (1)–(3), where, in this case, it has been considered that the laser can rotate with respect to the connecting line between camera and laser.

(1) $$x=\frac{a\cdot d}{f\cdot \text{cot}(\phi )-a}$$

(2) $$y=\frac{b\cdot d}{f\cdot \text{cot}(\phi )-a}$$

(3) $$z=\frac{f\cdot d}{f\cdot \text{cot}(\phi )-a}$$

where (*a, b*) are the coordinates in the focal plane, *d* is gap between the laser and the camera, *f* is the camera focal length, and *φ* is the laser angle with respect to the base line.

Distance computation in a classical approximation, usually consists of a loop of sequential instructions. Initially a frame is acquired by the camera, then there is a search through the sensor matrix looking for the pixels that exceed a certain threshold. Finally distances are calculated for each selected pixel. This process is repeated for each frame. A whole image search must be performed for each frame, this being an inherent sequential process.

### 3.2. System Calibration

**Figure 4.**
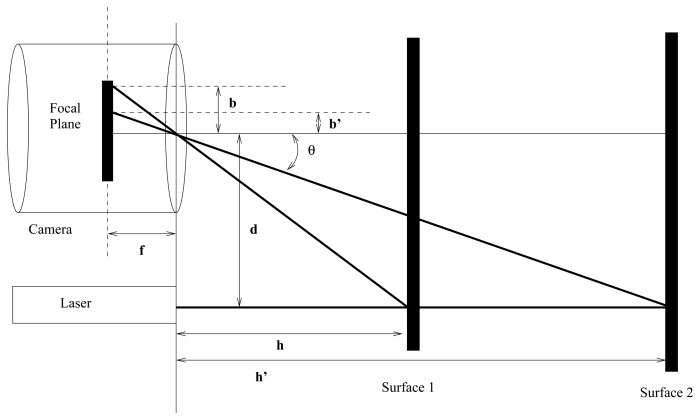
Simplified triangulation for one axis.

Equations (1)–(3) return accurate values when all the system variables are known. Nevertheless, if these parameters are not known with high precision, or if high accuracy is not a key factor in the experiment, as in this case, it is very common to use simplified formulae. Those approximations usually give good enough results. Figure 4 depicts a simplified representation of triangle equivalences already shown in Figure 3.

Taking into account Figure 4 it is possible to state:

(4) $$\frac{b}{d}=\frac{f}{h}$$

where *b* is the distance between the illuminated pixel and the image center, *d* is the laser-camera gap, *f* is the focal distance, and finally *h* is the distance to be measured. From Figure 4 it is also possible to rewrite Equation (4) as:

(5) $$h=\frac{d}{\text{tan}(\theta )}$$

Equation (5) shows the expression for the distance *h* between the system laser-camera and the surface to be measured. It can be obtained from *d*, the laser-camera gap, and the angle *θ*. This angle can be obtained as a function of the displacement in the image plane (in pixels). In this way, for a linear model the angle *θ* can be expressed as:

(6) θ=pfc⋅rpp+ρ

where *pfc* is the distance in pixels from the considered pixel to the image center, *rpp* are the radians per pixel, and *ρ* is a parameter useful for alignment error compensation. Consequently, from Equations (5) and (6) the following can be obtained:

(7) $$h=\frac{d}{\text{tan}(\text{pfc}\cdot \text{rpp}+\rho )}$$

*pfc* can be obtained easily from the image, but *rpp* and *ρ* must be obtained through a calibration process. With this aim, different measurements of pixel average displacement in the image plane (*pfc_i_*) were obtained for well-known and equally spaced distances *h_i_*. These values follow the equation:

(8) $$pfc_{i}\cdot \text{rpp}+\rho =\arctan\left(\frac{d}{h_{i}}\right)$$

With these data, and through an adjustment by least squares, the coefficients *rpp* and *ρ* can be obtained. Consequently, Equation (7) will give the scanned depth for *h* from the pixel distance to the center *pfc*.

If a higher grade polynomial interpolation is used, the coefficients must be obtained in a similar way.

(9) $$\theta =a_{3}\cdot pfc^{3}+a_{2}\cdot pfc^{2}+a_{1}\cdot \text{pfc}+a_{0}$$

**Figure 5.**
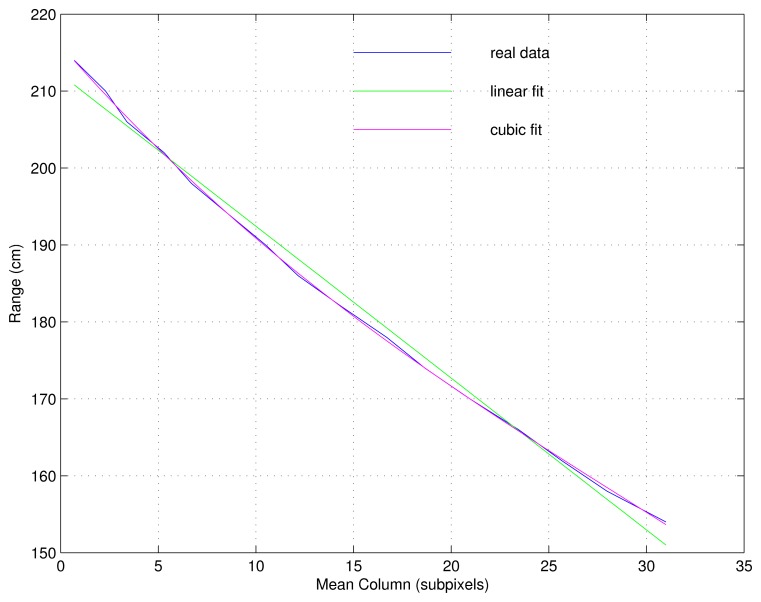
Linear and cubic adjustments for the relation distance/displacement.

Figure 5 shows both a linear and a cubic adjustment for the relation distance/displacement. The latter was selected as a trade-off between complexity and accuracy. The MSE (Mean Squared Error) is 0.31 cm for a grade 3 polynomial, *versus* MSE = 1.63 cm for a linear fit. Higher grades were discarded because they do not significantly improve the results, and they increase the complexity considerably.

The key factor for measurement accuracy is related with the minimum physical distance that generates a pixel variation in the image plane. When the range of detectable distances may vary among several meters, more than a few considerations must be taken into account. The use of special optical lenses to improve the laser beam convergence makes no sense. That is true since the lenses can be useful only for a very narrow range of distances, and the right lenses for a distance may blur the laser for others. The consequence, from a practical point of view, is that the laser thickness in the image plane can vary depending on the distance. In our system the option adopted was to compute the average value of active contiguous columns, weighed by its grey level. With this solution it is possible to obtain a subpixel resolution finding the value of a column which corresponds to a certain distance. The expression for computing the average column *pfc* for the *m-th* row is:

(10) $$pfc_{m}=\frac{\sum_{i=c_{i}}^{c_{f}}i\cdot P_{i}}{\sum_{i=c_{i}}^{c_{f}}P_{i}}$$

where *c_i_* and *c_f_* are the initial and final columns respectively with non-zero grey levels, and *p_i_* is the grey level of the *i-th* pixel, with 1 ≤ *m, f, i* ≤ 32.

To summarize, to compute the physical distance that corresponds to the *m-th* pixels row in the focal plane, which stand for a given *y* coordinate in the physical space, the Equation (10) must be used. Once the equivalent average column *pfc_m_* has been obtained, this value is replaced in the polynomial interpolation Equation (9) whose coefficients have been computed previously for a given range of distances.

With regard to the 32 × 32 SCD sensor in particular, the configuration angles of $\phi =\frac{\pi }{2}$ and $\theta =\frac{\pi }{2}$, a distance between the laser and the camera of *d* = 160 mm and not taking into account distortions due to camera optics, the detection physical range obtained was 62 mm in height, (*y* coordinate ranging from 0 to 62 mm), and 600 mm in depth, (*z* coordinate varying from 1,540 mm to 2,140 mm).

## 4. Experimentation

The scanning setup mounted on the pan-tilt camera gave good results for both cameras, the Firefly and the SCD. Nevertheless, it was necessary to rethink the experiment for two reasons:

1. The low resolution for both systems was a problem. Initially the servo motors needed to move as quickly as possible to guarantee variations in the sensor array plane. This low resolution meant the servo motors were not fast enough, compared to the acquisition speed.
2. The SCD camera prototype size, bigger than the Firefly camera, caused problems with the pan-tilt head due to its inertia.

**Figure 6.**
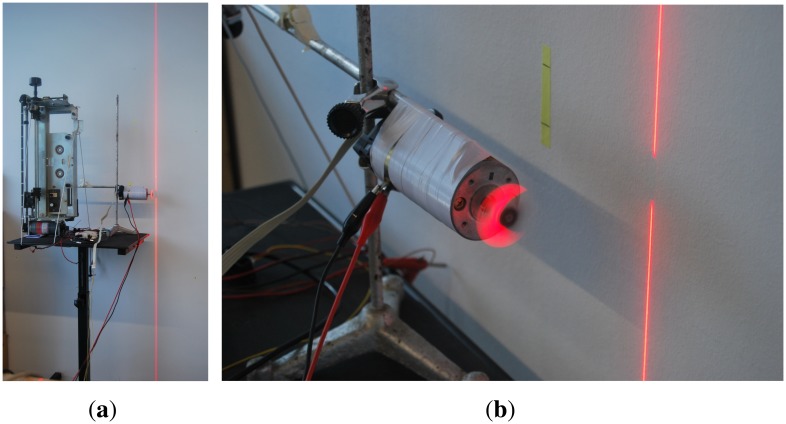
Laser beam projection into the helix. (**a**) Laser beam projection; (**b**) Detail of the moving helix.

For these reasons, instead of generating and acquiring scenes at high speed by rotating the entire system, it was decided to leave the camera-laser set static. Instead, a helix with four blades, each 2 cm long, attached to the axis of a DC (Direct Current) motor was used as the mechanism for generating high-speed changes. The speed of the motor is controlled by a computer and it is measured through an encoder. The high-speed movements are generated within its detection band. Distances in the *z* coordinate are in the range of 1,540 mm to 2,140 mm. Figure 6a shows the column laser projection achieved with the lenses. The detail of its incidence into the helix is shown in Figure 6b.

The static 3D reconstruction yield is the same for both systems, because all the static parameters of both systems have been chosen so as not to differentiate them in static scenes; this includes spatial resolution, grey level noise, and the 3D reconstruction method, which is the same for both systems. This has been done in this way because the aim of this work is to compare both strategies in the time/frequency domain. The SCD and the classical approaches yield the same results in static or low frequencies scenes if they have the same static parameters, and also if the SCD camera delivers at least as many pixels so as to cover the laser beam projection. These conditions have been met in all experiments; nevertheless, it is possible to reduce the number of pixels per frame of the SCD camera below the minimum necessary to recover the laser beam, without noticeable illumination profile degradation. This is because the SCD algorithm uses present and past information to recover the illumination profile with no extra processing, since the new results are obtained by modification of the last calculated value instead of calculating a new one for each frame [20].

**Figure 7.**
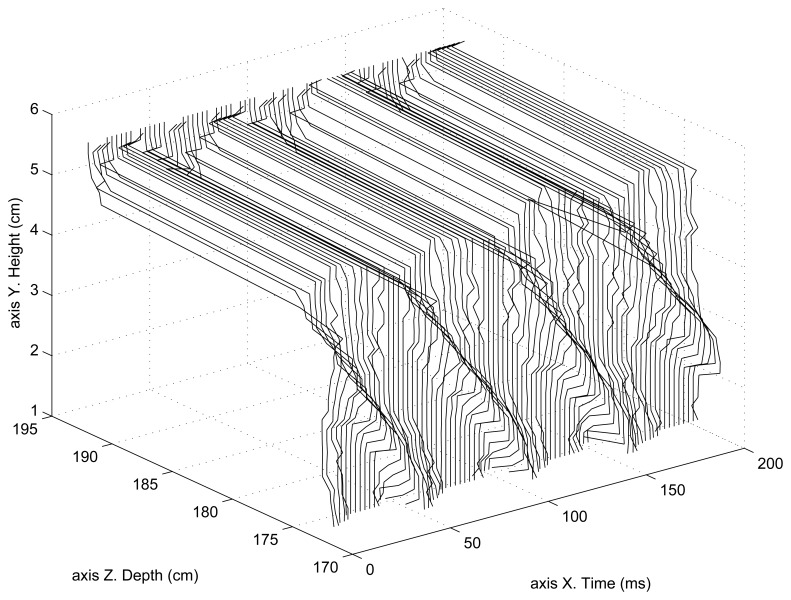
Rotating helix 2D reconstruction as a function of time.

Figure 7 shows the helix 2D reconstruction as a function of time. In this case, the helix is moving at 5 rps (revolutions per second). Since the helix has four blades, it generates a 20 Hz periodical movement. At these low frequencies, the 3D reconstruction (2D in this case) of both compared systems (SCD and classical) is the same, since the static parameters of both systems are the same as well as the 3D reconstruction method. This figure shows the upper part of the helix because the bottom part was becoming mixed with part of the upper section making it difficult to distinguish the front and back of the 3D surface (2D reconstruction plus time). This figure shows that the position of the rotating helix is located at roughly 175 cm from the camera, while the back wall is located at approximately 195 cm.

### 4.1. Classical Processing Scanner

The helix motor axis was placed in the detection range of the scanner ≈1,785 mm. Then, consecutive frames were acquired, being processed in a classical manner, as stated in Section 3.1. The rotation helix movement means that each of its blades approaches and departs periodically. In this way, the experiment would be equivalent to the scanning of an oscillating surface, with constant frequency and amplitude.

Different angular velocities in the motor helix were set, to try to experimentally confirm the theoretical frequency limit detection of this system. The acquisition plus processing time in the system was calculated for different angular velocities. The theoretical limitation, if the helix were to define a perfect sinusoidal surface, would be two samples (or frames) per revolution, as stated by the Nyquist–Shannon theorem.

**Figure 8.**
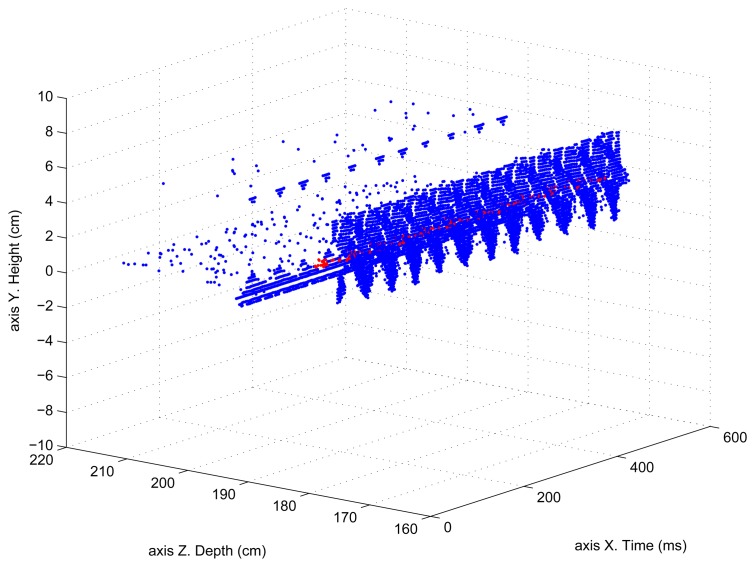
Helix depth and height as a function of time using classical processing with a 5 rps motor axis rotation speed.

250 frame sequences were acquired to determine this acquisition time. The total acquisition plus processing time was 607.52 ms, processing an average of 46 pixels/frame. With this data, the time needed for acquiring and processing one frame was 2.43 ms, with an average number of ≈11,500 processed pixels per second. The motor axis was initially rotating, in this first experiment to determine the processing time, at 5 rps. Figure 8 shows part of the acquired sequence. Each blue dot corresponds to a pixel which has contributed to the helix average position (red dots).

It must be noted that an angular velocity of 5 rps in the motor axis is equivalent to a surface angular velocity of 20 rps since the helix has 4 blades. In the case of the initial experiment, there were 20.8 frames per revolution, which gave an oversampled system.

The acquisition and processing time per sample, or frame, was 2.43 ms, as has been already stated. In a perfect periodical surface, 2 samples per revolution would be needed, which gives a time of 4.86 ms as the minimum period for acquiring and computing those samples. The inverse of this quantity gives an ideal maximum helix rotation speed of roughly 200 rps. In reality, as will be shown experimentally later, this frequency limit is a little bit lower.

**Figure 9.**
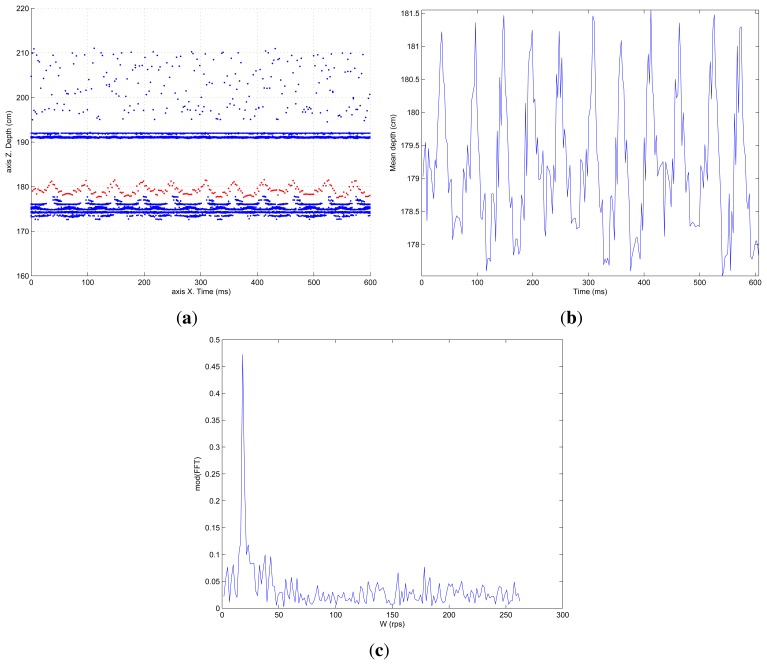
Depth map, average oscillation and FFT for 20 rps helix movement. Classical processing. (**a**) Depth map; (**b**) Average oscillation; (c) FFT.

The period, or other information such as frequency, oscillation amplitude, *etc.*, can be seen more clearly if only the *Z* coordinate is represented as a function of time. That has been done in Figure 9a, where the pixels (blue dots) that have been computed and that contribute to the average surface value (red dots) are shown. In Figure 9b the depth oscillation as a function of time can be seen more clearly. These values correspond perfectly to the real values with an average error of ≈5 mm.

A more straight forward way of showing the periodicity of a certain signal is to compute the FFT (Fast Fourier Transform). Figure 9c shows the FFT computed for the average depth value shown in Figure 9b. The frequency detection peak can be clearly seen in this figure centered at 20 rps.

**Figure 10.**
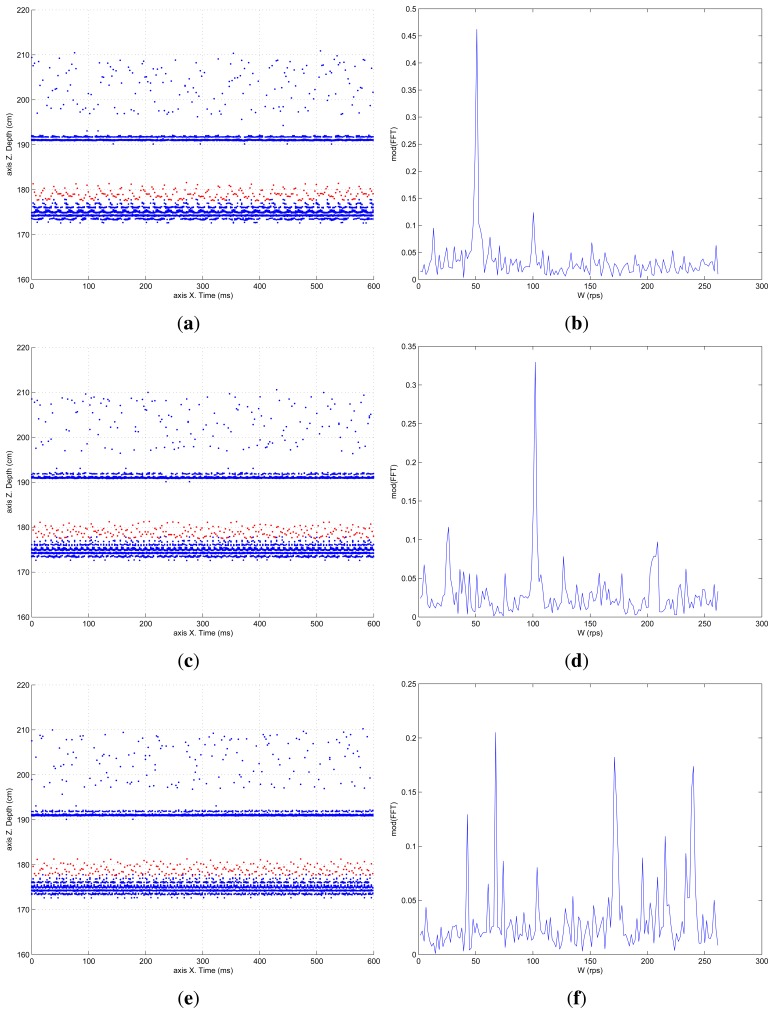
Depth map and average oscillation for 50, 100 and 175 rps helix movement. Classical processing. (**a**) Depth map for 50 rps; (**b**) FFT (Fast Fourier Transform) for 50 rps; (**c**) Depth map for 100 rps; (**d**) FFT for 100 rps; (**e**) Depth map for 175 rps; (**f**) FFT for 175 rps.

This experiment, with the classical camera and processing, was repeated increasing the helix rotation speed, trying, experimentally, to confirm the theoretical limitations. Figure 10, shows the pixel depth map and the computed FFT, for helix movements of 50, 100 and 175 rps. Graphics corresponding to the theoretical limit frequency of 200 rps have been omitted since they only showed noise. The FFT detects the surface frequency roughly at 175 rps, as can be seen in Figure 10b, although there are more peaks in the graphic. Frequencies above 175 rps show that the scanner is not able to detect anything similar to what should be an oscillating surface. Thus, it can be concluded that the experimental limit is lower than the theoretically calculated one. This fact can be explained by the fact that the helix blade movement does not generate a perfect periodical surface. There are discontinuities and imperfections that affect this detection limit, which is constrained by the classical sequential and processing approach of the system.

### 4.2. Selective Change Driven Scanner

With the SCD sensor the frame concept is no longer meaningful. The processing system receives the *n* pixels that have changed most. This parameter can be configured by software, and gives a powerful flexibility to the system. The number of pixels to be processed, and thus the processing time, can be adjusted taking into account the temporal restrictions. Moreover, not only are the number of pixels delivered to the processing system reduced, but the number of intermediate computations can also be reduced if a data-flow style programming [21] is used. These advantages have not been taken into account in this application, since no intermediate data have been stored and thus no time for updating them has been saved. In the scanner application the reduction in the number of acquired and delivered pixels can be extremely useful. Only the pixels that have changed their illumination level will be sent out and processed, reducing the limitations imposed by the Nyquist–Shannon sampling theorem.

With regard to the scanner application, it is expected that pixels with a greater variation would be those corresponding to the column illuminated by the laser. There would also be some of the neighboring columns which could be partially illuminated. Even taking that into account, it is clear that not all the pixels in the matrix need to be sent out and processed. A small percentage of pixels are going to be enough. Many experiments concerning the quantity of pixels that typically were illuminated by the laser image in the sensor plane were carried out. This quantity was determined to be between 40 and 50 pixels, so the camera software was programmed to deliver out the 45 pixels that changed most (4% of the total array). Most of the pixels of the sensor plane presented small grey level changes, but these were not caused by the direct laser illumination, so for this application it is a good idea not to take them into account.

Based on the above, a delivery rate of 45 pixels per integration time was selected, setting this integration time to 1 ms, that is approximately 22.2 μs per pixel. With these values, measurements using the same experiments performed with the standard camera, changing the angular velocities of the servomotor, were carried out. Depth maps and FFT figures for 20, 50 and 100 rps have been omitted in this paper since they show that the SCD system works as well as the classical one.

**Figure 11.**
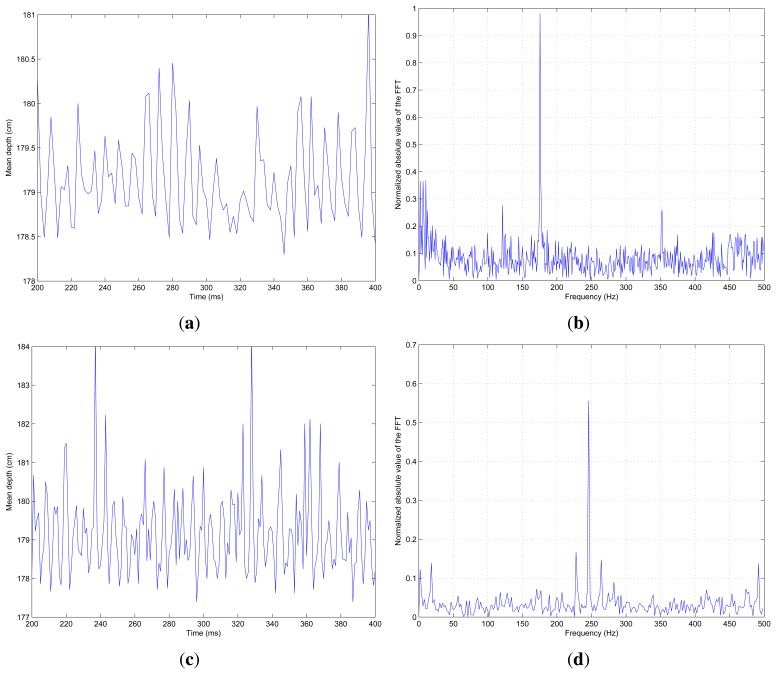
Mean depth and FFT for 175 and 240 rps helix movement. SCD processing. (**a**) Average depth for 175 rps; (**b**) FFT for 175 rps; (**c**) Average depth for 240 rps; (**d**) FFT for 240 rps.

Figure 11a shows the average depth of the helix for an oscillating surface of 175 rps and Figure 11b shows the computed FFT for these values. It is possible to see how the detection capability for the same helix speed has been improved. The SCD scanning system shows perfect frequency detection, while the classical scanning was not so good. The detection problems with the standard camera shown in Figure 10f, are now clearly surpassed.

Additionally, the helix was accelerated until it reached 200 rps (the theoretical limit for the classical system) and even beyond this. Figure 11c shows the measured average depth for the generated surface at 240 rps. Figure 11d shows the related FFT with perfect frequency detection. The experiment, with a surface oscillation of 240 rps, generated from a motor axis speed of 60 rps, was the highest frequency used. It was not possible, due to the motor limitations with the attached helix, to achieve higher frequencies. Nevertheless, the perfect frequency detection shown in Figure 11d suggests that the frequency could clearly be increased. Taking into account that the processing time for 45 pixels was negligible (9 μs) compared to the acquisition time of 1 ms, and two samples for the detection of a perfect oscillating surface are needed, the theoretical limit would be 500 rps. In fact this limit could be even greater, reducing the SCD acquisition time.

Table 1 briefly compares both scanner prototypes. This table shows that one of the most important differences is the bandwidth requirements, since the bandwidth required by the SCD system is one order of magnitude less than the classical system; this is one of the main advantages of SCD vision. More frame rate is also available for the SCD system compared to the classical one. Also, the acquisition time of the SCD system is reduced as shown in Table 1.

## 5. Conclusions

A comparison between two scanner prototypes has been presented, one with a classic camera and another with an SCD sensor. The SCD sensor has a resolution of 32 × 32 pixels, so the resolution of the conventional camera has been reduced to the same number of pixels for comparative purposes. The main idea has been to explore the limitations of a laser-based 3D scanning system for both approaches.

A helix in the axis of a computer controlled DC motor was mounted in order to obtain a very fast moving surface. Experimentally it has been shown how the classical approach is much slower than the SCD approach. In the classical way of computing which pixels have to be taken into account, the whole sensor matrix must be searched, using the grey values for computing the illuminated average column. This inherently sequential process introduces a delay that unavoidably limits the functioning speed of the scanning system.

On the other hand, an SCD sensor only delivers the pixels that have changed most, from the last read out frame. Moreover, those pixels are ordered by the absolute magnitude of their change. In this way, it is possible to just read a fixed number of pixels, which will be the most significant ones. In the case of the laser projected in a surface, the number of pixels that will be illuminated and that can contribute to the depth computation can be estimated. Only this number of pixels is read out, and this in fact is similar to the number of pixels that contribute to the depth computation in the classical approach. The computation has been significantly speeded-up because there is no processing waste due to having to read and select throughout the whole matrix.

As a final conclusion, it has been shown experimentally how a Selective Change-Driven sensor can speed-up the use of a surface scanning system. The selective reduction of information performed by this kind of sensor, sending only the pixels that have changed, can speed-up these systems.

Research in SCD sensing and processing is being continued on several fronts: new sensors, new processing architectures and new applications. Despite the fact that the sensor presented has demonstrated the usefulness of the SCD philosophy, the sensor resolution can be considered low and this fact limits its use in practical applications. A new SCD sensor with a higher resolution (64 × 64 pixels), and better performance is being developed at the moment. More complex applications will take advantage of this philosophy of acquiring and processing visual information with higher resolution. On the other hand, SCD sensing is not only limited to the way of acquiring and delivering out visual information. This technique is related with data-flow processing, a technique of processing information that optimizes the resources used, processing only data that have changed. A changing pixel only triggers the instructions that depend on it. Following these ideas, it is possible to implement a custom data-flow architecture to process only changing information. This architecture is being developed in an FPGA (Field-Programmable Gate Array) board. Finally, besides the improvement of the SCD-based scanning system, any image processing application, which benefits significantly from data reduction, could be speeded-up with this kind of sensors.

## Figures and Tables

**Table 1.** Classical *vs.* SCD comparison table.

	**Classical Approach**	**SCD Approach**
Bandwidth requirements	412 Kb/s (@ 243 fps)	44 Kb/s (@ 1000 fps)
Frame rate	243 fps	1,000 fps
Highest detected frequency	175 rps (scarcely)	240 rps (and more)
Acquisition+processing time (per pixel)	53.8 μs	22.2 μs
